# Canine Chondrodysplasia Caused by a Truncating Mutation in Collagen-Binding Integrin Alpha Subunit 10

**DOI:** 10.1371/journal.pone.0075621

**Published:** 2013-09-25

**Authors:** Kaisa Kyöstilä, Anu K. Lappalainen, Hannes Lohi

**Affiliations:** 1 Research Programs Unit, Molecular Neurology, University of Helsinki, Helsinki, Finland; 2 Department of Veterinary Biosciences, University of Helsinki, Helsinki, Finland; 3 Department of Molecular Genetics, Folkhälsan Institute of Genetics, Helsinki, Finland; 4 Department of Equine and Small Animal Medicine, Faculty of Veterinary Medicine, University of Helsinki, Helsinki, Finland; University of Bergen, Norway

## Abstract

The skeletal dysplasias are disorders of the bone and cartilage tissues. Similarly to humans, several dog breeds have been reported to suffer from different types of genetic skeletal disorders. We have studied the molecular genetic background of an autosomal recessive chondrodysplasia that affects the Norwegian Elkhound and Karelian Bear Dog breeds. The affected dogs suffer from disproportionate short stature dwarfism of varying severity. Through a genome-wide approach, we mapped the chondrodysplasia locus to a 2-Mb region on canine chromosome 17 in nine affected and nine healthy Elkhounds (p_raw_ = 7.42×10^−6^, p_genome-wide_ = 0.013). The associated locus contained a promising candidate gene, cartilage specific integrin alpha 10 (*ITGA10*), and mutation screening of its 30 exons revealed a nonsense mutation in exon 16 (c.2083C>T; p.Arg695*) that segregated fully with the disease in both breeds (p = 2.5×10^−23^). A 24% mutation carrier frequency was indicated in NEs and an 8% frequency in KBDs. The *ITGA10* gene product, integrin receptor α10-subunit combines into a collagen-binding α10β1 integrin receptor, which is expressed in cartilage chondrocytes and mediates chondrocyte-matrix interactions during endochondral ossification. As a consequence of the nonsense mutation, the α10-protein was not detected in the affected cartilage tissue. The canine phenotype highlights the importance of the α10β1 integrin in bone growth, and the large animal model could be utilized to further delineate its specific functions. Finally, this study revealed a candidate gene for human chondrodysplasias and enabled the development of a genetic test for breeding purposes to eradicate the disease from the two dog breeds.

## Introduction

The human skeletal dysplasias are rare disorders of varying severity that present with abnormalities in the skeletal patterning, development, growth and maintenance [1], [2]. Majority has an autosomal recessive or dominant inheritance pattern [3]. In the most recent classification effort, over 450 distinct entities were placed in the broad group of human genetic skeletal disorders, and altogether 226 skeletal dysplasia genes were reported [3]. The known causative genes, which explain 316 of the characterized skeletal disorders [3], encode a variety of different proteins, including extracellular matrix (ECM) components, enzymes, ion channels, signal transducers and transcription factors [1], [2], [4], [5].

Three general subgroups of skeletal dysplasia are commonly recognized, although the clinical characteristics can overlap. Osteodysplasia and chondrodysplasia are generalized disorders of the bone and cartilage tissues, whereas the dysostoses affect individual bones or group of bones [1]. Primary osteodysplasias are characterized by altered bone mineral density and chrondrodysplasias by abnormal endochondral ossification, which affects the linear growth of bones and typically results in disproportionate short stature [6], [7]. Bone formation through endochondral ossification takes place in the epiphyseal growth plates of the long bones. The cartilaginous growth plates are composed of ECM and linear columns of differentiating chondrocytes that are organized into zones of resting, proliferating, mature and hypertrophied cells, subsequently replaced by trabecular bone [8], [9]. Disruptions in genes and proteins that affect growth plate physiology have direct effects in endochodral ossification and lead to various pathologies [4], [9]. A significant group of chondrodysplasia-causing genes constitute those that code for cartilage ECM proteins, such as cartilage oligomeric matrix protein (COMP), proteoglycans aggrecan and perlecan and several different collagens [10]–[13]. Other examples of causative genes include fibroblast growth factor receptor 3 (*FGFR3*) and parathyroid hormone 1 receptor (*PTHR1*), which encode receptors that regulate growth plate chondrocyte differentiation and proliferation [14]–[17], and a sulfate transporter (*SLC26A2*), which codes for a sulfate transporter that maintains adequate sulfation of ECM proteoglycans [18]–[20].

In addition to humans, inherited chondrodysplasia occurs in the pure-bred dog [21], [22]. The population structure of modern dog breeds is characterized by high inbreeding, severe bottlenecks and isolation, all of which have increased the incidence of simple and complex inherited diseases [23], [24]. As a result of the unique population history, linkage disequilibrium is relatively long within breeds, enabling genome-wide association studies (GWAS) to be performed using small sample numbers [25], [26]. Inherited chondrodysplasia has been described in several breeds, including the Alaskan Malamute [27]–[29], Norwegian Elkhound [30], Miniature Poodle [31] Samoyed [32], Labrador Retriever [33], [34], Scottish Deerhound [35], English Pointer [36], Great Pyrenees [37] and the Irish Setter [38]. Autosomal recessive inheritance has been proposed in Alaskan Malamutes [39], Great Pyrenees [37] and Irish Setters [38]. The causative mutation is known in few breeds but the majority of the phenotypes have an unknown molecular genetic cause. Recently, a collagen type XI (*COL11A2*) mutation was associated with mild chondrodysplasia in Labrador Retrievers [40], and a sodium/sulfate cotransporter (*SLC13A1*) mutation was shown to cause severe dwarfism in the Miniature Poodle breed [41]. Furthermore, an oculo-skeletal dysplasia in Samoyeds and Labrador Retrievers has been linked to recessive collagen type IX mutations (*COL9A2* and *COL9A3*, respectively) [42]. In addition to being an anomalous phenotype, chondrodysplasia occurs in some breeds as a fixed, breed-defining trait [43]–[47]. In a number of these breeds, the short-limb phenotype has been shown to be due to an expressed fibroblast growth factor 4 (*FGF4*) retrogene [47].

The Norwegian Elkhound (NE) is a Nordic hunting breed that suffers from disproportionate short-limbed dwarfism. The condition, which is caused by a generalized dysfunction in the endochondral ossification process, has been clinically and histologically characterized in the 1980’s [30]. The radiographic findings in affected NEs include curved front limbs, carpal valgus, shortening of vertebral bodies, delayed ossification of carpal bones and increased metaphyseal width and flaring [30]. Growth plate histology reveals several changes, which include unusual wide bars of ECM, disorganized columnar structure and atypical large chondrocytes with altered morphology [30]. The genetic background of the phenotype has not been studied before.

We report here our efforts to identify the gene mutation behind NE chondrodysplasia. We successfully mapped the disease locus through a genome-wide approach and identified a fully segregating nonsense mutation. We also show that the same recessive mutation segregates with a similar short-limbed dwarfism in another Nordic hunting breed, the Karelian Bear Dog (KBD), supporting its causative nature. Our present findings have both scientific and practical implications on bone biology and canine breeding programs, respectively.

## Results

### Pedigree Analysis and Radiographic Examinations Reveal a Recessive Condition with Variable Skeletal Changes

**Figure 1:**
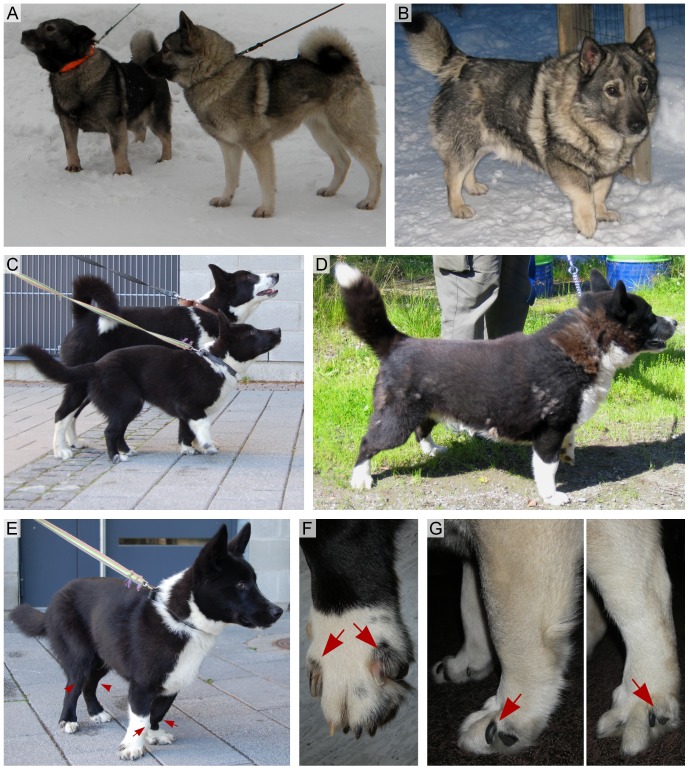
Chondrodysplastic and normal Norwegian Elkhounds and Karelian Bear Dogs. (A) A 5-year-old affected female Norwegian Elkhound with chondrodysplasia (left) and a 3-year-old unaffected female Norwegian Elkhound (right). The height at withers was 42 cm for the affected and 48 cm for the unaffected dog. (B) A 7-year-old affected male Elkhound with a height at withers of 38 cm. (C) A normal 5-month-old male Karelian Bear Dog together with its severely affected and significantly smaller male littermate. (D) An adult, less severely affected Karelian Bear Dog that is actively used in hunting. (E) The 5-month-old affected male puppy has prominent bilateral carpal valgus (arrows) and knock knees (genu valgus) (arrowheads). The muscles of the pelvis and thigh are underdeveloped due to severe hip dysplasia. (F) The left forepaw of the 5-month-old affected puppy. Outer digits are abnormally short (arrows). (G) The left forepaw (left) and the left hind paw (right) of an adult affected Elkhound. Similarly to the Karelian Bear Dog, the outer digits are abnormally short in this affected dog (arrows).

**Figure 2:**
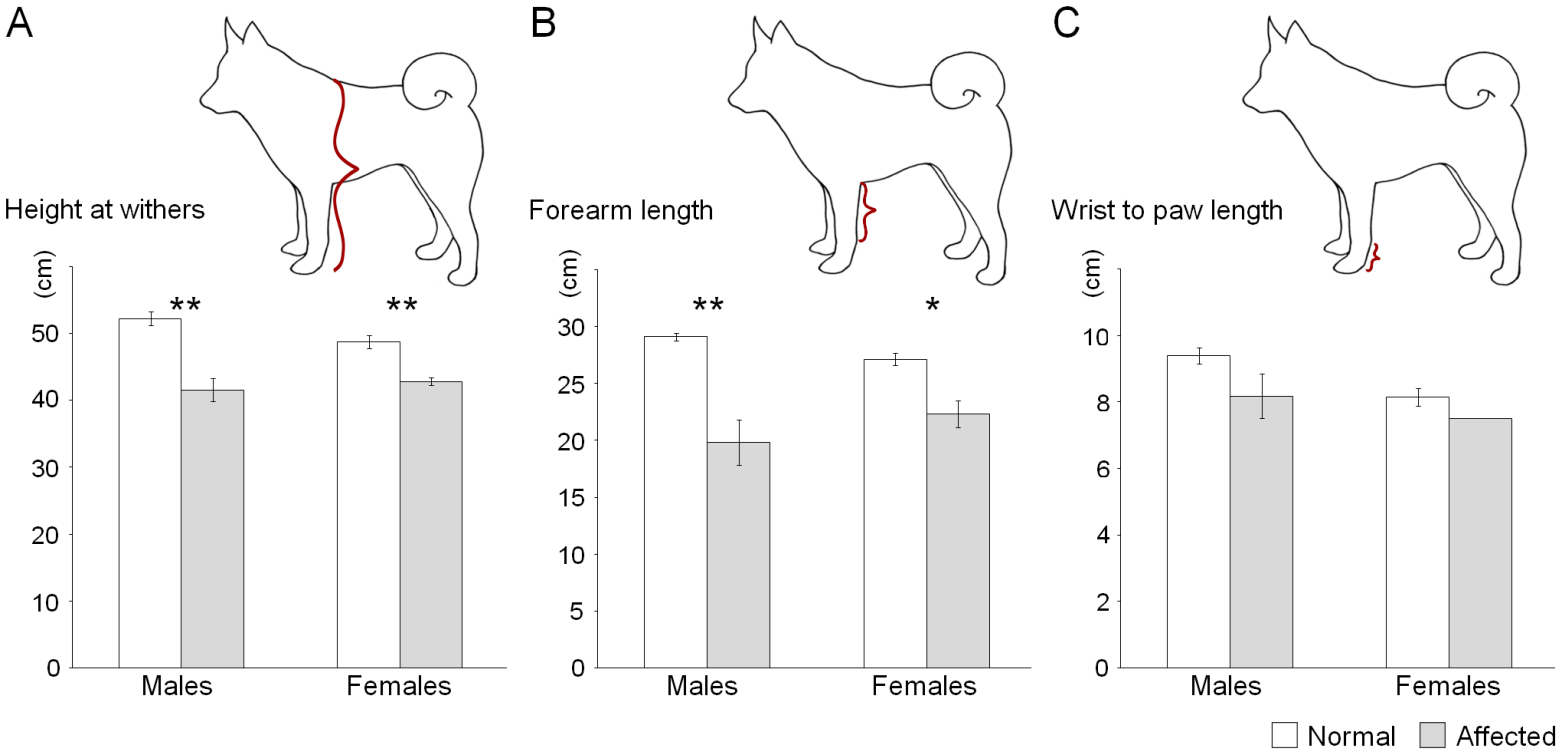
Size differences between chondrodysplastic and normal Norwegian Elkhounds. Bar plots show the difference of means concerning (A) height at withers, (B) forearm length and (C) wrist to paw length. Error bars represent the standard error of the mean. Measurements were taken from 14 unaffected and 6 affected males, and from 11 unaffected and 3 affected females. The wrist to paw length was measured in two affected females only. *p≤0.01, **p≤0.001.

Our research group was approached by Finnish NE breeders that were concerned about the relatively high occurrence of short-legged dwarf dogs in the breed. The affected NEs were viable with normal cognition but had approximately 10 cm shorter limbs than normal (**Figure 1A and B**). In order to verify the dwarf phenotype, NE owners were asked to provide three size parameters as a part of the sampling effort: height at withers, forearm length and the length from wrist to paw (metacarpal and carpal bones). The size measurements were obtained from nine adult affected NEs, comprising six males and three females and from 25 unaffected adult NEs, comprising 14 males and 11 females (**Table 1**). The mean height at withers and the length of the forearm differed significantly between the affected and unaffected groups in both males (p_withers_ = 0.001 and p_forearm_ = 0.000, two-tailed Mann-Whitney U-test) and females (p_withers_ = 0.000 and p_forearm_ = 0.002, two-tailed t-test) (**Figure 2A and B**). No statistically significant difference was found in the length of metacarpal and carpal bones in either sex (**Figure 2C**).

**Table 1:** Body length measurements in affected and healthy Norwegian Elkhounds.

Sex	Status	N	Height at withersMean±SD (cm)	ForearmMean±SD (cm)	Wrist to pawMean±SD (cm)
Males	Unaffected	14	52.3±1.3	29.1±1.3	9.4±0.9
	Affected	6	41.6±4.2	19.8±4.9	8.2±1.6
Females	Unaffected	11	48.7±1.4	27.1±1.8	8.1±0.9
	Affected	3	42.8±1.0	22.3±2.1	7.5±NA*

* Measured only in two affected females.

**Figure 3:**
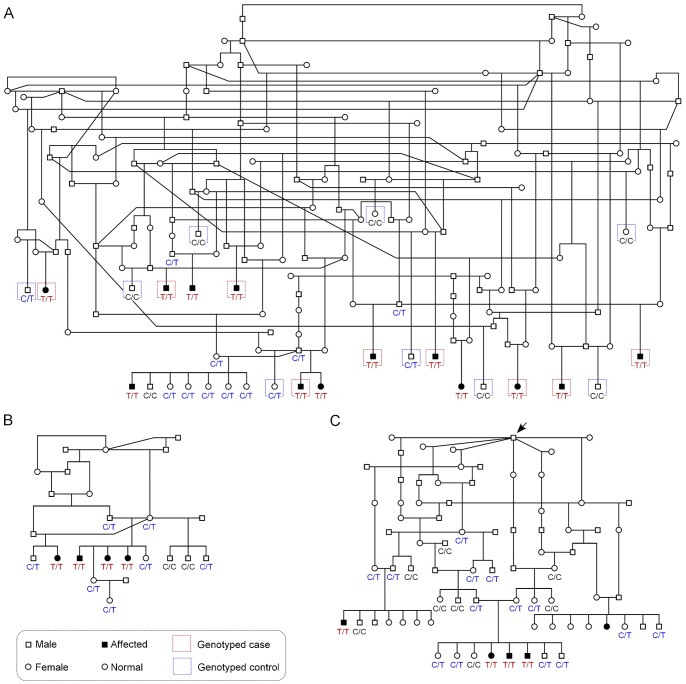
Chondrodysplasia pedigrees are consistent with autosomal recessive inheritance. (A) A pedigree established around the affected Norwegian Elkhounds from Finland. Samples and phenotype information were obtained from all siblings in one litter only, otherwise the phenotypes of full siblings of affected dogs were not known. Denoted are the nine cases and controls that were genotyped using the canine SNP-chip. (B) A pedigree drawn around four affected Norwegian Elkhounds from the United States. (C) A pedigree of the chondrodysplasia phenotype in Karelian Bear Dogs. All affected Karelian Bear Dogs have a single popular sire as a common ancestor (arrow). In all three pedigrees, the recessive c.2083C>T mutation shows full segregation with the chondrodysplasia phenotype. Genotypes are marked with red (T/T), blue (C/T) and black (C/C).

Blood samples were obtained from altogether 13 adult affected NEs from Finland and four adult affected NEs from the United States. The pedigrees established around the known affected NEs suggested an autosomal recessive mode of inheritance (**Figure 3A and B**). All the parents of affected dogs were normal height dogs and both genders were affected.

As the study had progressed with NEs, it came to our attention that a similar type of dwarfism occurred in another Nordic breed, the KBD (**Figure 1C–E**). At least three different litters with affected dogs were reported in the KBDs. All affected litters could be traced back to a single male (arrow) that lived in the 1980’s and sired over 450 puppies in its lifetime (**Figure 3C**). There was a strong suspicion within the breed that this popular sire had not been a purebred KBD but a NE-KBD mix, which suggested that the two breeds might share a causative mutation. As in NEs, the mode of inheritance in KBDs was consistent with a recessive model since the parents were unaffected (**Figure 3C**). Altogether four affected KBDs were sampled for this study, three of which were 5-month-old juvenile dogs from the same litter and the fourth was an adult affected dog.

A 3-year-old affected male NE and affected and healthy 5-month-old male KBD littermates (**Figure 1C**) volunteered for a clinical study and were referred to a radiographic examination. On outward appearance, the affected NE had short stature but no ambulatory difficulties, whereas the juvenile affected KBD preferred sitting down to standing up as it suffered from hip dysplasia and subsequent underdevelopment of pelvis and thigh muscles (**Figure 1E**). In addition, the affected KBD had abnormally developed digits, a condition, which was also present in a few affected NEs (**Figure 1F and G**).

**Figure 4:**
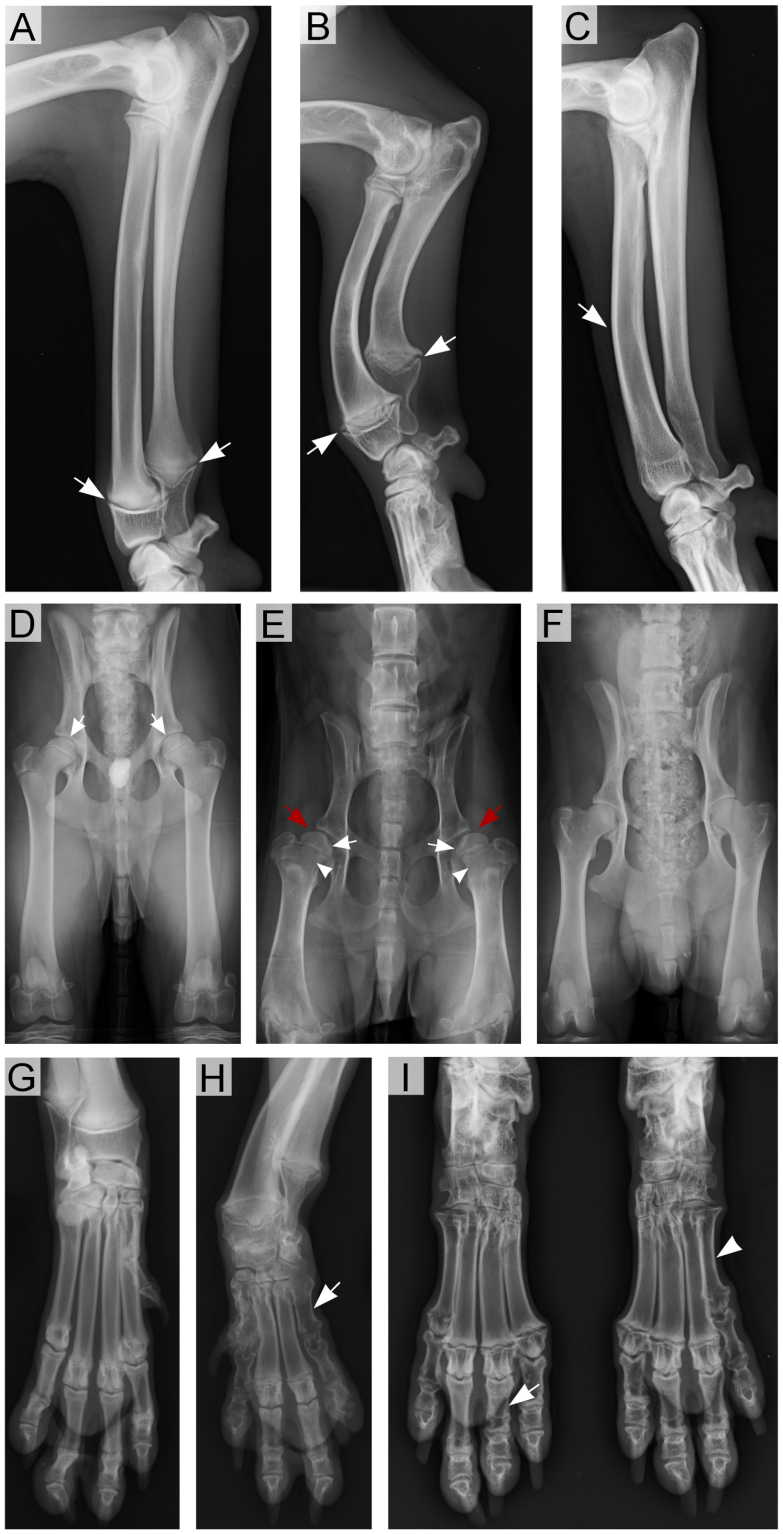
Radiographic findings in affected dogs. (A) The forearm of an unaffected 5-month-old male Karelian Bear Dog has narrow and even growth plates (arrows). (B) The forearm of a severely affected 5-month-old male Karelian Bear Dog with markedly short and bowed radius and ulna. The growth plates are wide and irregular and metaphyseal flaring can be observed (arrows). (C) The forearm of a 3-year-old affected male Norwegian Elkhound. The radius is slightly bowed cranially (arrow). (D) Normal hip joints of an unaffected 5-month-old Karelian Bear Dog. The femoral head sits in its correct position (arrows). (E) Abnormal hip joints of a 5-month-old affected Karelian Bear Dog. The femoral heads are misshapen (white arrow), femoral necks are abnormally short (arrowhead) and the joints are subluxated (red arrow). (F) Normal hip joints of a less severely affected 3-year-old Norwegian Elkhound. (G) Normal metacarpal bones and digits of an unaffected 5-month-old Karelian Bear Dog. (H) Distal forelimb of an affected 5-month-old Karelian Bear Dog with a very short fifth metacarpal bone (arrow). (I) Distal hind limbs of an affected 5-month-old Karelian Bear Dog. Wide growth plates and metaphyseal flaring are apparent. The proximal phalanx of the third digit of the right hind limb (arrow) and the fifth metatarsal bone of the left hind limb (arrowhead) are abnormally short. Dogs in images (A) and (B), (D) and (E) and (G)–(I) are littermates.

Radiographs of limbs, spine and skull were obtained from all three dogs. The adult dwarf NE was skeletally less affected than the juvenile KBD (**Figure 4**). The radii of the affected NE were slightly bowed cranially (**Figure 4C**), the epiphyses were wider and the thorax shallower than normally but otherwise the skeleton appeared to be within normal limits. The radiological findings in the affected KBD were more pronounced. Compared to the narrow and even growth plates of the normal littermate, the growth plates of the affected KBD were wide and irregular and the metaphyseal regions were flared (**Figure 4A and B**). The limbs, especially the forearms (radius and ulna), were considerably shortened and curved cranially (**Figure 4A and B**). The femoral heads and necks were misshapen and the hip joints subluxated (**Figure 4D–F**). The length of metacarpi and proximal phalanges was not constant and varied in and between the limbs (**Figure 4G–H**). The spine and skull appeared more normal even if the vertebral epiphyses were somewhat widened.

### Genetic Analyses Map the Chondrodysplasia Locus to CFA17 and Reveal a Nonsense Mutation in the *ITGA10* Gene

**Figure 5:**
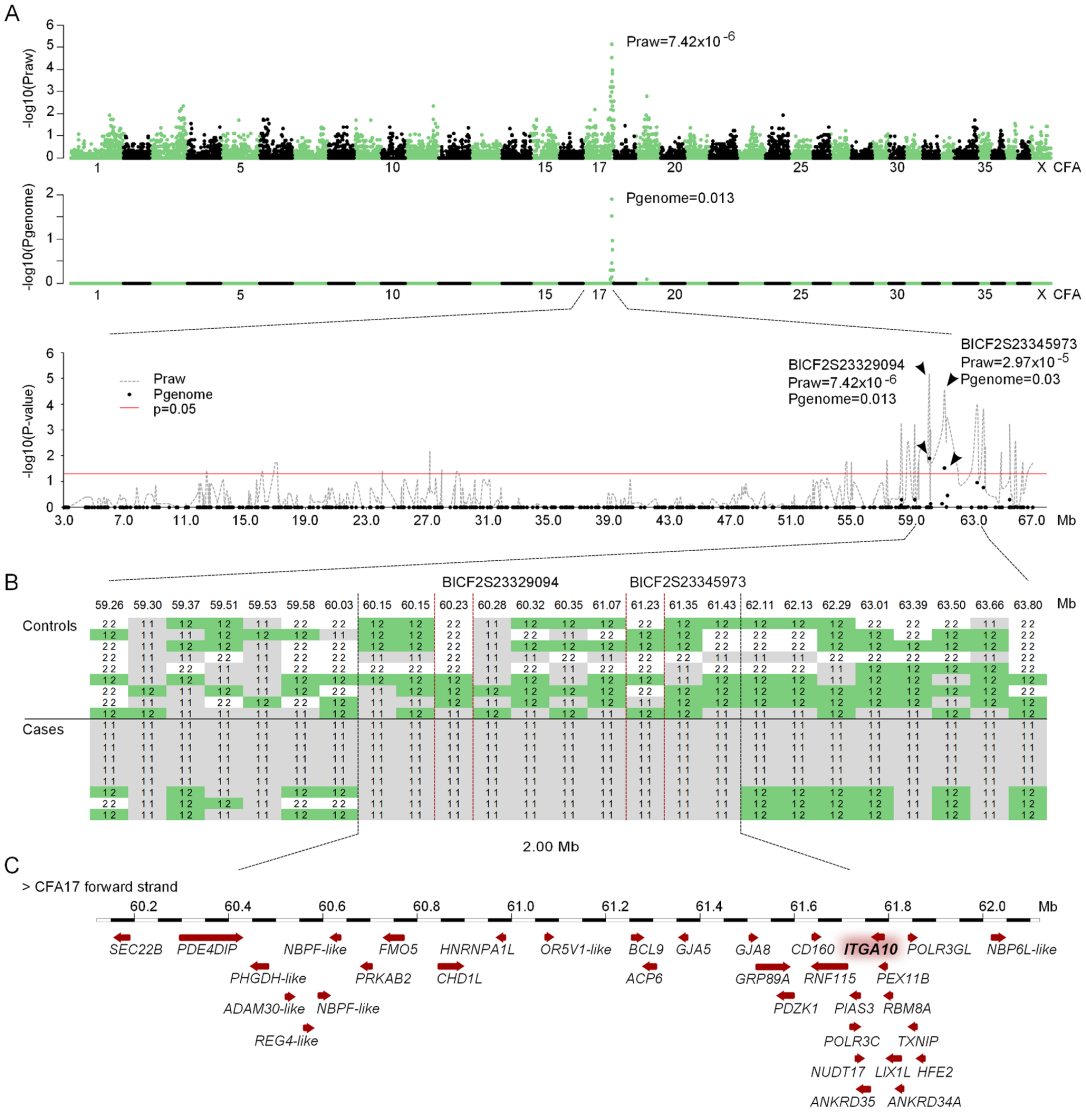
Results of genome wide association analysis. (A) The chondrodysplasia locus maps to CFA17. The Manhattan plots show both nominal and permutated p-values of the Fisher’s exact test across all chromosomes. A close-up of CFA17 shows two SNPs, BICF2S23329094 and BICF2S23345973 that reach genome-wide significance after permutation testing. (B) Genotypes at the CFA17 associated locus reveal a shared 2-Mb haplotype block in the affected dogs. (C) The critical region contains 33 genes, including *ITGA10*, which was selected as a primary candidate gene due to its known expression in the growth plate chondrocytes and involvement in the endochondral ossification process.

A GWAS was performed to map the chondrodysplasia locus in the NE breed. Nine affected and nine control NEs were genotyped by using Illumina’s 22K canine SNP chip. A case-control association analysis revealed a genome-wide significant association on CFA17 (p_raw_ = 7.42×10^−6^, p_genome-wide_ = 0.013; Fisher’s exact test) (**Figure 5A**). At the associated locus, all affected dogs shared a 2-Mb homozygous haplotype block that spanned from 60 to 62 Mb (CanFam2.0 assembly) (**Figure 5B**) and contained altogether 33 genes (**Figure 5C**). Integrin subunit alpha 10 (*ITAG10*) was considered the best candidate for mutation screening since it was known to be expressed on growth plate chondrocytes [48], and had been shown to affect bone growth in an *Itga10*-null mouse model [49].

**Figure 6:**
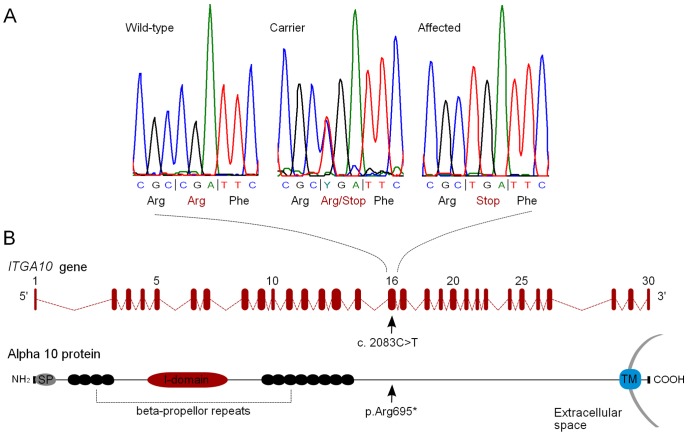
A homozygous nonsense mutation in *ITGA10*. (A) Chromatograms of the mutation position in a wild-type, a carrier and an affected dog. (B) A schematic representation of *ITGA10* gene structure and of α10 protein domains. The protein coding sequence of the canine *ITGA10* gene is composed of 30 exons, and the c.2083C>T change is positioned on exon 16. The α10-subunit is a single pass transmembrane protein with a small cytosolic domain. The largest part of the protein is located in the extracellular space. The nonsense mutation p.Arg695* is positioned approximately in the middle of the α10-subunit. SP = signal peptide, TM = transmembrane segment.

All 30 *ITGA10* exons were sequenced in two affected and two control NEs. The controls comprised an obligate carrier parent and a half-sibling to a dwarf dog. The sequencing revealed altogether four intronic and five exonic variants (**Table 2**). Four exonic variants were synonymous changes but one was a non-synonymous C to T substitution in exon 16 (c.2083C>T) (**Figure 6A and B**), which introduces a premature stop codon (p.Arg695*) to the encoded protein, integrin subunit alpha 10 (α10) (**Figure 6B**). The c.2083C>T change segregated with the phenotype in the four sequenced NEs. The two affected dogs were homozygous (T/T) for the change, the obligate carrier parent heterozygous (C/T), and the half-sibling had a wild-type genotype (C/C). The other eight identified variants did not segregate with the phenotype but were homozygous in all four sequenced dogs. The segregation of the c.2083C>T allele was followed further in affected and healthy NEs (n = 58) (**Table S1**). In Finnish NEs, all affected dogs (n = 13) were homozygous for the T allele and all obligate carriers (n = 4) were heterozygous. Six full-siblings and 22 other relatives had either a heterozygous or a wild-type genotype (**Figure 3A**). Similarly, in an additional NE family cohort from the United States, all affected NEs (n = 4) were homozygous for the T allele and two known obligate carrier parents were heterozygous. Five out of seven unaffected family-members were heterozygous and two were wild-type dogs (**Figure 3B**).

**Table 2:** A summary of the variants found in the mutation screening *ITGA10* exons in two affected and two healthy Norwegian Elkhounds.

Position	Variant	Amino acid change	Segregation
Intron 2	c.164+47C>A	–	No
Intron 3	c.274+97T>C	–	No
Exon 6	c.519T>C	p.Asp173Asp>silent	No
Intron 8	c.909+8T>G	–	No
Exon 9	c.1044T>C	p.Asp348Asp>silent	No
Exon 14	c.1734T>C	p.His578His>silent	No
Exon 16	c.1938C>G	p.Val646Val>silent	No
Exon 16	c.2083C>T	p.Arg695*>nonsense	Yes
Intron 26	c.3108+69T>G	–	No

The following *ITGA10* reference sequences were used in naming variants: XM_845262.1 (mRNA), XP_850355.1 (protein) and NC_006599.2 (genomic).

We then examined whether the c.2083C>T mutation is also present in the affected KBDs. Genotyping of a cohort of 68 KBDs revealed a full segregation between the nonsense mutation and the chondrodysplasia phenotype also in this breed (**Table S1**). All affected KBDs (n = 4) were homozygous for the T allele and all parents of affected dogs (n = 4) were heterozygous. Six out of eight full-siblings carried the mutated allele, whereas two had a wild-type genotype (**Figure 3C**). Out of 52 unaffected relatives, ten carried the T allele and 42 were homozygous for the wild-type allele. Collectively, these results revealed a full segregation between the c.2083C>T mutation and the chondrodysplasia phenotype in both breeds (p = 2.5×10^−23^, n = 126). As an additional support, the mutation was not found in 192 dogs from 12 other breeds (**Table S1**).

Carrier frequency of the c.2083C>T mutation was 24% in a cohort of 156 randomly selected Finnish NEs and 8% in a population sample of 287 KBDs. Curiously, all except for one heterozygous KBD (36 out of 37) could be traced back to the popular sire that was allegedly a NE-KBD mix (**Figure 3C**). Out of all wild-type KBDs, 66% (194 out of 295) could be traced back to this same sire.

### The Integrin Alpha 10 Protein is Absent in the Affected Tissue

**Figure 7:**
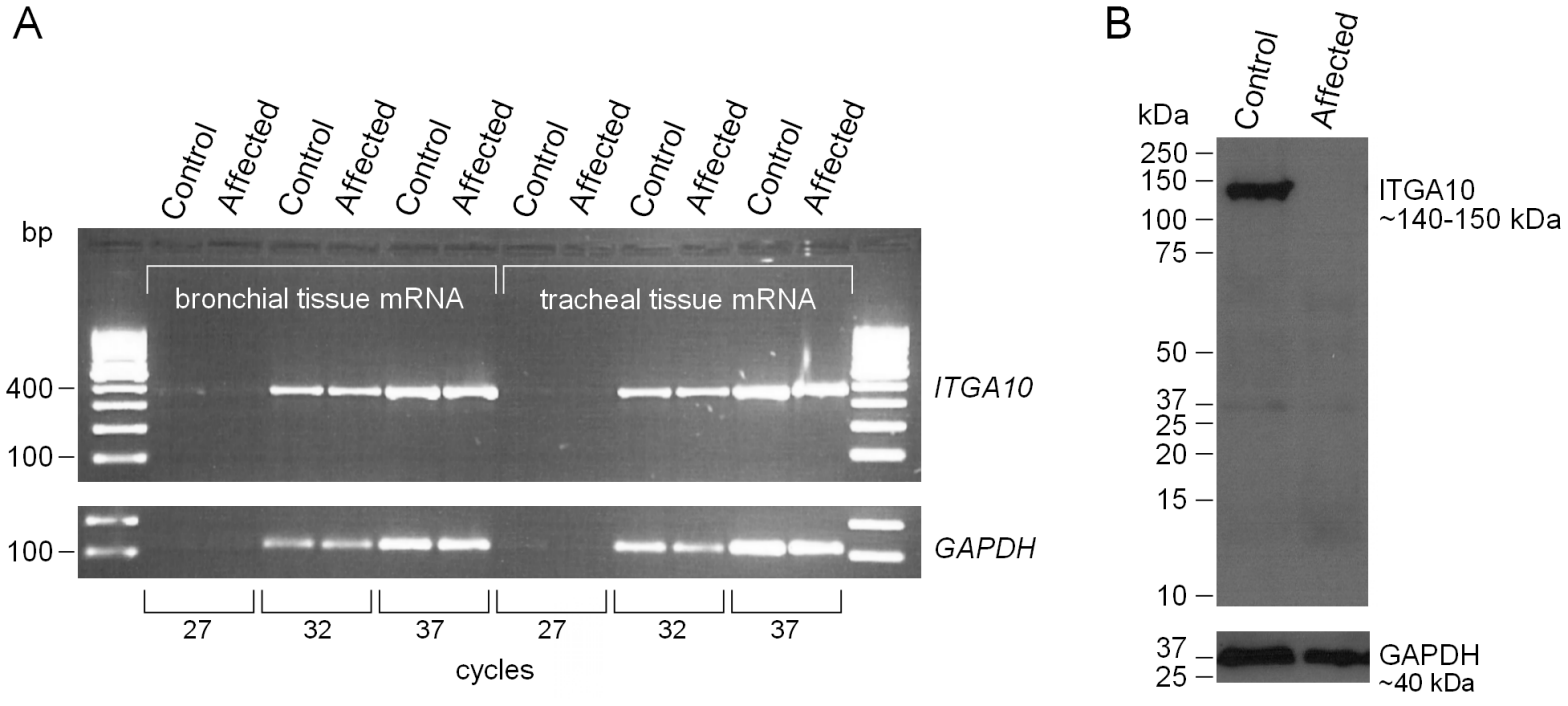
*ITGA10* expression on the RNA and protein level. (A) Semi-quantitative analysis of *ITGA10* mRNA expression in bronchial and tracheal tissue samples of an affected NE and an unaffected Australian Kelpie dog. PCR reactions were performed using three cycle numbers, 27, 32 and 37. Amplification of mRNA fragments was roughly equal in both dogs, which indicated that the mutated transcript is stable and not targeted for nonsense mediated decay. (B) A western blot analysis of ITGA10 protein expression. A polyclonal anti-ITGA10 antibody was probed against the total protein lysates from tracheal tissue samples of the affected NE and the unaffected Australian Kelpie. The full-length ITGA10 protein was detected in the unaffected control dog but not in the affected dog. GAPDH was used as a loading control.

We next studied the potential effects of the c.2083C>T *ITGA10* mutation at the mRNA and protein levels. Since the encoded protein, integrin α10-subunit, is specifically expressed in cartilage-containing tissues [48], we used cartilaginous bronchial and tracheal tissue samples from one affected and one control dog. Sequencing of the full length *ITGA10* mRNA verified the presence of mutation in the affected dog and confirmed the exon-intron boundaries of the reference sequence (XM_845262.1). In addition, we detected alternative splicing of exon 24 in the tissue samples of both the affected and control dog. This finding is in accordance with a previous study that indicated differential splicing of human and murine *ITGA10* transcripts [50]. However, different exons were reported to be involved in human and mouse; exon 25 was alternatively spliced out in humans, and in mice, transcription of exon 26 is extended to the following intron resulting in a truncated transcript [50]. Unexpectedly, a semi-quantitative PCR analysis did not reveal observable changes in *ITGA10* transcript levels between the affected and control samples (**Figure 7A**), which suggested that the mutated transcript is not readily targeted for nonsense-mediated mRNA decay (NMD) in the bronchial and tracheal tissue samples. We examined this further by using real-time quantitative PCR, which provides a more sensitive quantification method. The experiment was performed on five different tissues collected from one affected and one wild-type dog. In the lung and bronchial tissue, the results were suggestive of a reduced *ITGA10* expression in the affected dog but the other tested tissues, trachea, small intestine and spleen, indicated an opposite effect (**Figure S1**). This could be suggestive of tissue-specific NMD, however, more samples would be needed to confirm this.

We then proceeded to study the effects of the nonsense mutation at the protein level. The *ITGA10* gene product, integrin α10-subunit, assembles into a αβ-heterodimeric cell surface receptor. The identified p.Arg695* nonsense mutation is predicted to shorten the 1165-amino-acid α10-subunit by 471 residues from the carboxyl-terminal end, eliminating the cytosolic tail, the transmembrane domain and a significant proportion of the extracellular protein bulk (**Figure 6B**). To assess the protein level effects, we performed a western blot analysis using tracheal tissue lysates and a polyclonal anti-ITGA10 antibody. A strong signal was present in the control tissue whereas the protein was absent in the affected dog, and no truncated protein product was detected (**Figure 7B**). This indicates a complete loss of protein function in the affected dog.

## Discussion

We have performed a comprehensive genetic study to identify the cause of the chondrodysplasia phenotype that was described in the Norwegian Elkhound 30 years ago [30]. Our results indicate that the disease is caused by a recessive nonsense mutation (c.2083C>T, p.Arg695*) in the *ITGA10* gene. The nonsense mutation is predicted to eliminate nearly half of the encoded integrin subunit α10 protein, and we show that the protein is absent in the affected tissue. As an independent confirmation of causality, we demonstrate that the same mutation shows full segregation with a corresponding phenotype in a genetically different breed, the KBD. The canine chondrodysplasia phenotype implicates an essential role for *ITGA10* in endochondral ossification and reveals a candidate gene for similar conditions in other species, including human.

*ITGA10* represents an excellent candidate gene for chondrodysplasia. The encoded integrin subunit α10 belongs to the integrin family of proteins [51], which constitute a group of bidirectional cell surface receptors that mediate cells’ interactions with the surrounding ECM and other cells [52]–[55]. Altogether eight α- and 18 β-subunits have been described in vertebrates, and these combine into 24 different αβ-heterodimers that have distinct ligand binding and signaling properties [52], [53], [56]. The integrin α10-subunit assembles into an α10β1 heterodimer [51], which belongs to the collagen receptor subgroup of integrins, together with three other β1 integrins, α1β1, α2β1 and α11β1. All four possess a specific collagen-binding I-domain in their α-subunit [57]–[61]. The α10β1 heterodimer was originally identified as a collagen type II-binding integrin in bovine chondrocytes [51]. In addition to collagen type II, α10β1 has been shown to bind laminin and other collagen types as well, and may show preference to the non-fibrillar collagen types IV and VI [62]. Prior to this study, no spontaneous disease-causing mutations have been reported for either *ITGA10* or for the β1-subunit gene *ITGB1*.

A previous study in mice indicated that the α10 protein is primarily expressed in cartilage chondrocytes [48]. The prenatal expression of α10 in the developing murine cartilage coincides with chondrogenesis and collagen type II expression [48]. During post-natal development, α10 is expressed by chondrocytes throughout the growth plate cartilage [48]. The major collagen constituent of the cartilage ECM is collagen type II, and other less abundant collagen components include types IX, X and XI [63]–[65]. Mutations in several different collagen genes are known to cause various forms of chondrodysplasia and other skeletal disorders [12], [66]. Mutations in the type II procollagen gene *COL2A1* alone cause at least ten different forms of skeletal dysplasia [3], [67]. While many disease mutations are recognized in collagen genes, they have been rare in collagen-binding receptors. Chondrodysplasia-causing mutations has been reported only for a discoidin domain receptor 2 (DDR2) [68]–[71], which is a receptor tyrosine kinase that regulates chondrocyte differentiation and proliferation during endochondral ossification [70], [72], [73]. Importantly, our study associates the second collagen-binding receptor gene to a naturally-occurring inherited chondrodysplasia.

The precise signals that are mediated by the α10β1 receptor during endochondral ossification are unclear but studies in induced α10- and β1-deficient mouse models indicate roles in matrix fibril assembly and chondrocyte proliferation [49], [74]. Mice that lack the integrin α10-subunit gene have been reported to suffer from mild chondrodysplasia and to present with a slight reduction in the length of the long bones [49]. The skeleton of the α10-null mice appeared otherwise normal, apart from a reduction in the length of tibia and femur to 93-90% when compared to wild-type mice [49]. The skeletal phenotype of the affected dogs is considerably more pronounced. The initial characterization of the NE dwarf phenotype in the 1980’s revealed a generalized skeletal disorder with significant metaphyseal changes in all growth centers, especially at the distal metaphyses of the radii and ulnae [30]. The affected NEs had significantly shorter long bones than unaffected dogs, bowed front limbs and valgus deformity at the carpi. Furthermore, ossification of carpal cuboid bones was delayed, and the vertebral bodies were affected, which caused a reduction in the length of the torso [30]. Our present clinical findings are in accordance with the previous phenotypic characterization. However, the radiographic examination of a severely affected KBD revealed significantly misshapen femoral necks and heads, which was not reported before.

Similar to the overall skeletal changes, the histological changes are milder in the α10-null mice than in affected dogs but there are some shared features [30], [49]. The gross morphological appearance of the α10-deficient murine growth plates was reported not to differ from wild-type mice. However, a more detailed examination revealed some slight changes, including a reduced number of cell layers in zone of chondrocyte hypertrophy and some disorganization of chondrocyte columns in the zone of chondrocyte proliferation [49]. More pronounced changes were described in affected NEs [30]. In 10-week-old affected animals, the zone of chondrocyte proliferation was decreased to half in width, contained unusual wide bars of ECM and had a disorganized column formation. Moreover, the disorganization continued to the columnar structure of the hypertrophic and degenerating zones, and also to the trabeculae of the metaphyseal spongiosa [30]. Interestingly, both mice and dogs developed misshapen, spherical chondrocytes, when the normal appearance of the proliferating chondrocytes is flattened [30], [49]. Similar atypical chondrocyte morphology has been described in β1-deficient growth plates as well [74]. A targeted inactivation of the *ITGB1* gene in murine cartilage, and the subsequent loss of all β1-integrins, has been shown to result in frequent perinatal lethality and severe chondrodysplasia with markedly abnormal growth plate morphology [74]. Both α10- and β1-deficient murine growth plates show reduced collagen fibril density and a cell cycle defect [49], [74]. In accordance with this, an abnormal accentuation of ECM fibril structure and a decreased width of the proliferative zone were reported in the growth plates of dwarf NEs [30]. Taken together, the skeletal defect and histological changes in the α10-deficient mice offer strong support for the causality of the now identified canine mutation. However, the severity of the canine phenotype indicates a more critical role for the α10β1-receptor during endochondral ossification in larger sized mammals.

We examined the effects of the identified nonsense mutation both at mRNA and protein levels. Unexpectedly, we were unable to detect evidence of reduced *ITGA10* mRNA expression in all tested tissues of the affected dog, which might be indicative of tissue-specific NMD. The NMD is a mechanism that protects cells from the possible detrimental effects of truncated polypeptides that result from premature translation termination codons [75]. According to a proposed general mechanism, those premature stop codons that are at least 50–55 nucleotides upstream from a 3′-exon-exon junction are a target of NMD [76]. The p.Arg695* nonsense change in exon 16 of canine *ITGA10* should therefore elicit the NMD response. However, the process of NMD is still not completely understood and exceptions to the above mentioned rule have been reported [77]–[80]. There is also evidence of tissue and developmental stage specific regulation of NMD [81]–[84]. Unfortunately, we had very limited tissue material available (one case and one control), to confirm the possible tissue-specific NMD. Moreover, we were not able to collect growth plate cartilage tissue from affected and unaffected adolescent dogs, which would have been optimal to determine transcript levels relative to the phenotype in question. It is a possibility that the mutated transcript escapes mRNA surveillance, leading to the translation of a truncated product. However, a truncated protein would likely be unstable. Accordingly, our immunoblotting experiment revealed an absence of the full length ITGA10 protein, which indicates a loss of function, and is in accordance with the recessive canine phenotype. Since heterozygous carrier dogs do not exhibit a skeletal phenotype, a dominant negative mutation caused by an aberrant truncated protein product would be unlikely. However, there is a possibility that the polyclonal antibody we used in the immunoblotting experiment did not recognize a truncated N-terminal protein, and therefore further studies would be needed to clarify the matter.

In strong support of pathogenicity, the nonsense mutation identified in NEs, was found in another breed, the KBD, that had the same clinical manifestation. Moreover, the mutation segregated with the chondrodysplasia phenotype in a separate NE family cohort from the US. A high mutation carrier frequency of almost one in every fourth dog was recorded within the Finnish NEs, whereas the frequency was notably lower in the Finnish KBDs. The results of our carrier screening suggested that a single popular KBD sire, that was probably a part NE, if not introduced, at least highly enriched the frequency of the mutation in KBDs. A simple genetic test is now offered for NE and KBD breeds to help control the carrier frequencies and to eradicate the condition from the breeds.

Our results implicate the *ITGA10* gene as a plausible candidate gene for humans. Although the previously described α10-deficient mouse model already revealed a mild skeletal phenotype, the more pronounced chondrodysplastic changes in the larger spontaneous canine model make *ITGA10* a strong causative candidate in human disproportionate chondrodysplasias. Since the dog is more close to human in size and physiology, a phenotype of similar severity would be likely. Currently, the molecular genetic background is known for many human conditions but there are those, in which the causative genes are yet to be identified [3]. The radiographic and histological features of the affected NEs have been suggested to correspond to the changes found in human spondylometaphyseal dysplasias [30]. Given the lack of intermediate phenotypes in the heterozygous dogs and the likely loss of all *ITGA10* function in affected dogs, the corresponding human conditions would likely be recessive chondrodysplasias.

In summary, we identify a novel canine chondrodysplasia gene, *ITGA10,* which also represents a candidate gene for human chondrodysplasias. The canine phenotype provides additional evidence of the critical role of the α10β1 receptor in bone growth, and offers a large animal model for further functional studies. Finally, our findings have enabled the development of a genetic test for the affected breeds.

## Materials and Methods

### Ethics Statement

All animals used in this study were privately owned pet dogs, and the owners gave permission for their dogs to be used in the study. The research was approved by the Animal Ethics Committee at the State Provincial Office of Southern Finland (permits: ESLH-2006-08207/Ym-23 and ESHL-2009-07827).

### Animals and Samples

The entire study cohort comprised 214 Norwegian Elkhounds, 336 Karelian Bear Dogs and a control cohort of 192 dogs from 12 other breeds (**Table S1**). The Finnish NE cohort of 45 dogs included altogether 13 affected NEs from 12 different litters (**Figure 3A**). In one litter, samples were received from the affected dog, both parents and from all six unaffected littermates. Other samples included two obligate carrier parents and 22 more distant relatives. An additional NE family cohort of 13 dogs was received from the United States, comprising four affected dogs from two litters, two obligate carrier parents, six unaffected littermates and one other unaffected relative (**Figure 3B**). The KBD sample cohort of 68 dogs comprised four affected dogs out of two different litters, eight full siblings, four obligate carrier parents and 52 relatives. One KBD litter was covered fully, as both parents and all affected and healthy littermates were sampled (**Figure 3C**). Pedigrees were drawn around the affected dogs by using the GenoPro genealogy software (http://www.genopro.com/) and the Finnish Kennel Club’s pedigree registry KoiraNet (http://jalostus.kennelliitto.fi/). Population cohorts were used to estimate mutation carrier frequencies. In Finnish NEs, a population cohort of 156 unaffected dogs was randomly selected among those NE samples that had been collected for other research projects. In KBDs, a population cohort of 287 unaffected dogs comprised all those KBD samples that had been collected before the chondrodysplasia study was initiated.

Majority of research samples were collected as EDTA-blood. Three KBD samples and the NE family cohort from the United States were received as buccal swabs. DNA was extracted from the whole blood samples by using a semi-automated Chemagen extraction robot (Chemagen Biopolymer-Technologie AG) and the buccal swap samples were extracted by using the QiaAmp DNA Mini Kit (Qiagen). DNA concentrations were determined by using a ND-1000 UV/Vis Spectrophotometer (NanoDrop Technologies) and the samples were stored at −20°C until further use.

### Body Length Measurements

A set of three body measurements were collected from affected and normal NEs. Owners were asked to measure the height at withers, the length of the forearm from elbow to wrist and the length from wrist to paw (carpal and metacarpal bones) according to detailed instructions. Measurements were obtained from altogether 34 dogs, comprising 14 control males, six affected males, 11 control females and three affected females. The wrist to paw length was measured in two affected females only. Statistical analysis on length measurements were performed using PASW Statistics 18 software (IBM). The Student’s t-test was used when equality of variances was fulfilled (Levene’s test p was >0.05) and if not, the Mann-Whitney U-test was applied.

### Genome-wide Association Analysis

Nine affected and nine healthy NEs were genotyped using Illumina’s CanineSNP20 BeadChip of 22,362 validated SNPs. The nine affected dogs were from different litters. The potential confounding effects of population stratification were accounted for by using matched second-degree relatives (half-siblings) or more distant relatives as genotyping controls (**Figure 3A**). Genotype data was filtered using a minor allele frequency of >5%, SNP call rate of >95% and a sample call rate of >95%. A total of 7679 SNPs were removed for low minor allele frequency and 238 SNPs failed the missingness test. Since the total genotyping rate was 99.2%, no samples had to be removed for low sample call rate. No significant deviations were detected from the Hardy-Weinberg equilibrium with the threshold at p≤0.0001. After all filtering steps, 14,626 SNPs remained for analyses. The Fisher’s exact test was performed to calculate allelic association between cases and controls using software package PLINK [85]. Genome-wide significance was ascertained through phenotype permutation testing (n = 50,000).

### Mutation Screening

Canine *ITGA10* (XM_845262.1) sequencing primers (**Table S2)** were designed using Primer 3 (http://frodo.wi.mit.edu/primer3/). PCR reactions were performed in a 20 µl reaction volume that contained 20 ng of genomic DNA, 1×PCR buffer, 2 mM MgCl_2_, 0.2 mM dNTPs, 0.5 µM of forward- and reverse primers, 1 M betaine (Sigma-Aldrich) and 1 unit of Biotools DNA Polymerase. PCR products were run on a 1% agarose gel stained with *GelRed (Biotium, Inc)*. Sequencing reactions were carried out using a 3730×l DNA Analyzer (Applied Biosystems), and sequence data was analyzed by using Variant Reporter v1.0 program (Applied Biosystems). Applied Biosystems’ TaqMan chemistry and 7500 Fast Real-Time PCR instrumentation were used to genotype the control cohorts. The probe sequence for the wild-type allele was 5′-CACTCACAGAATCGGCGAT-3′ and 5′-CACTCACAGAATCAGCGAT-3′ for the mutated allele. Amplification primers were 5′-CTCCTGGCCGCTGGA-3′ and 5′-GGGCTGAGAGTTGCTTAGGA-3′, forward and reverse, respectively. The Taqman genotyping reactions were performed in a 10 µl reaction volume with 10 ng of genomic DNA, 1×TaqMan genotyping assay and 1×Genotyping Master Mix (Applied Biosystems).

### Tissue Samples

One 3-year-old affected male NE was euthanized on owner’s decision due to aggressive behavior towards humans and other dogs. Samples from various different tissues were collected immediately after euthanasia and stabilized in RNAlater (Ambion, Inc). The stabilized tissue samples were kept in −80°C until subsequent use. Control tissue samples were obtained from a 2-year-old male Australian Kelpie that was put down because of severe epileptic seizures.

### RNA Experiments

Total RNA was extracted from tracheal and bronchial tissue samples of one affected NE and one unaffected Australian Kelpie. RNA extraction was performed using the RNeasy Mini Kit (Qiagen). Concentration of RNA samples was determined by using a ND-1000 UV/Vis Spectrophotometer (NanoDrop Technologies), and equal amounts of RNA were reverse-transcribed into cDNA by using the High Capacity RNA-to-cDNA Kit (Applied Biosystems). The full-length *ITGA10* mRNA was sequenced from the cDNA samples by using primers that were designed to span multiple exons in order to control for genomic DNA contamination (**Table S2**). The mRNA sequencing reactions and data analysis was carried out as described in methods for mutation screening.

Semi-quantitative PCR and real-time *quantitative PCR (qPCR)* were used to study the levels of *ITGA10* mRNA in affected and control dog. In the semi-quantitative analysis, one of the mRNA sequencing primer pairs, ITGA10_mRNA_7 (**Table S2**), was amplified in equal amounts of the tracheal and bronchial cDNA samples. Amplifications were performed using three different cycle numbers (27, 32 and 37) to ensure that a logarithmic amplification phase was detected. Expression levels were evaluated on 1.5% agarose gel stained with GelRed (Biotium, Inc). *The q*PCR experiment was performed by using two different ITGA10 primer pairs (**Table S2**), and the obtained expression data was normalized against two house-keeping genes, GAPDH and YWHAZ. The PCR reactions were performed using Applied Biosystems’ 7500 Fast Real-Time PCR instrumentation and Roche’s FastStart Universal SYBR Green Master according to the manufacturers’ instructions. Triplicate samples were used for all reactions. The efficiencies of the qPCR reactions were calculated from a seven-point dilutions series and the relative expression differences were calculated using the comparative ΔΔCt-method [86].

### Protein Expression Analysis

Total protein lysates were prepared for a western blot experiment from tracheal tissue samples of one affected NE and one unaffected Australian Kelpie dog. The RNAlater preserved tissue samples were homogenized on ice using the Pierce T-PER Tissue Protein Extraction Reagent (Thermo Fisher Scientific) with Pierce Halt Protease Inhibitor Cocktail (Thermo Fisher Scientific) added to the extraction buffer. Pierce BCA Protein Assay Kit (Thermo Fisher Scientific) was used to determine protein concentration in order to load equal amounts of protein lysates for 9% SDS-PAGE and subsequent western blotting. A mouse polyclonal anti-ITGA10 antibody (Sigma-Aldrich #SAB1411763) was used as primary antibody in immunoblotting, together with a mouse monoclonal antibody for GAPDH (Invitrogen #39–8600). The polyclonal anti-ITGA10 antibody was raised against the full length ITGA10 and was therefore expected to recognize a possible truncated protein. The immunoblotting signals were detected by using a horseradish peroxidase-conjugated anti-mouse IgG antibody (GE Healthcare #NA931) and Pierce SuperSignal West Femto Maximum Sensitivity Substrate (Thermo Fisher Scientific).

## Supporting Information

Figure S1 **Relative expression levels of** ***ITGA10*** **mRNA.** The relative expression levels of *ITGA10* mRNA were studied in five different tissues using samples from one affected Norwegian Elkhound and one unaffected wild-type dog from another breed. Two primer pairs, (A) ITGA10_q1 and (B) ITGA10_q2, that were positioned on opposite ends of the canine *ITGA10* mRNA were used to determine relative expression levels. Two tested tissues (lung and bronchus) showed a clear decrease of *ITGA10* expression in the affected dog. The other tissues (trachea, small intestine and spleen) showed an increase in the *ITGA10* expression in the affected dog. Error bars represent the standard error of Ct-values.
(TIF)

Table S1 **Dogs genotyped for the** ***ITGA10*** **mutation.**
(XLSX)

Table S2 ***ITGA10*** **sequencing and qPCR primers.**
(XLSX)
