# Supplementary figures and images for: Canine Chondrodysplasia Caused by a Truncating Mutation in Collagen-Binding Integrin Alpha Subunit 10
Source: PLoS One. 2013 Sep 25;8(9):e75621. doi: 10.1371/journal.pone.0075621 (PMC3783422; doi:10.1371/journal.pone.0075621)

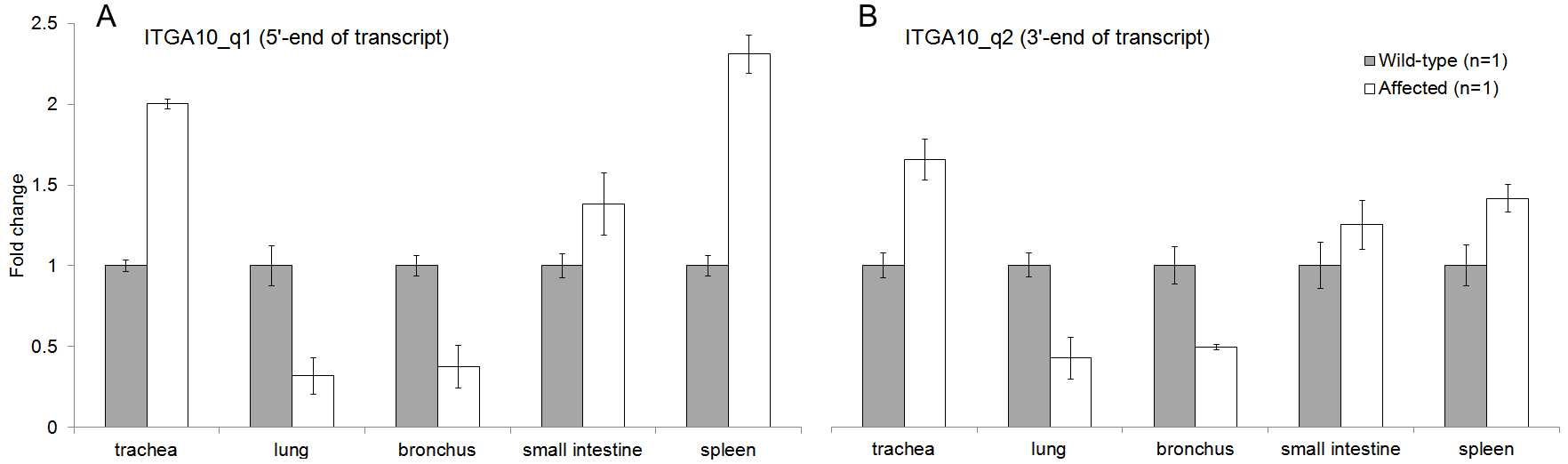

Supplement: Figure S1 — Relative expression levels of ITGA10 mRNA. The relative expression levels of ITGA10 mRNA were studied in five different tissues using samples from one affected Norwegian Elkhound and one unaffected wild-type dog from another breed. Two primer pairs, (A) ITGA10_q1 and (B) ITGA10_q2, that were positioned on opposite ends of the canine ITGA10 mRNA were used to determine relative expression levels. Two tested tissues (lung and bronchus) showed a clear decrease of ITGA10 expression in the affected dog. The other tissues (trachea, small intestine and spleen) showed an increase in the ITGA10 expression in the affected dog. Error bars represent the standard error of Ct-values. (TIF) [file pone.0075621.s001.tif]
